# Supplementary material for: Accessing Voluntary HIV Testing in the Construction Industry: A Qualitative Analysis of Employee Interviews from the Test@Work Study
Source: Int J Environ Res Public Health. 2021 Apr 15;18(8):4184. doi: 10.3390/ijerph18084184 (PMC8071335; doi:10.3390/ijerph18084184)
Supplement: Supplementary file 1 [file ijerph-18-04184-s001.zip › ijerph-1176120-supplementary/ijerph-1176120-supplementary/Supplementary file S1- COREQ.pdf]

Supplementary file S1: Consolidated criteria for reporting qualitative studies (COREQ): 32-item checklist

Topic and Item No.	Guide Questions/Description	Response
Domain 1: Research team and reflexivity		
Personal Characteristics		
1. Interviewer/facilitator	Which author/s conducted the interviews?	Sarah Somerset (SS)
2. Credentials	What were the researcher's credentials? E.g. PhD, MD	SS: PhD, Bsc, Mres
3. Occupation	What was their occupation at the time of the study?	SS: Research fellow
4. Gender	Was the researcher male or female?	Female
5. Experience and training	What experience or training did the researcher have?	SS: Experienced interviewer
Relationship with participants		
6. Relationship established	Was a relationship established prior to study commencement?	Researcher met the participants during recruitment
7. Participant knowledge of the interviewer	What did the participants know about the researcher? e.g. personal goals, reasons for doing the research	Participants knew that SS was a health researcher and that she was affiliated to the participating university
8. Interviewer characteristics	What characteristics were reported about the interviewer/facilitator? e.g. Bias, assumptions, reasons and interests in the research topic	Participants knew that SS was interested in the experiences of employees participating in the health checks
Domain 2: Study design		
Theoretical framework		
9. Methodological orientation and Theory	What methodological orientation was stated to underpin the study? e.g. grounded theory, discourse analysis, ethnography, phenomenology, content analysis	Thematic analysis
Participant selection		
10. Sampling	How were participants selected? e.g. purposive, convenience, consecutive, snowball	Convenience sampling of participants after participating in health check events
11. Method of approach	How were participants approached? e.g. face-to-face, telephone, mail, email	Participants were approached and recruited face to face at the health check events.
12. Sample size	How many participants were in the study?	426 participants
13. Non-participation	How many people refused to participate or dropped out? Reasons?	For the 88 participants declining interview, 50 (11.7%) were too busy, 13 (3.1%) experienced communication barriers and 25 (5.9%) did not give a specific reason.
Setting		

14. Setting of data collection	Where was the data collected? e.g. home, clinic, workplace	Workplace
15. Presence of non-participants	Was anyone else present besides the participants and researchers?	No
16. Description of sample	What are the important characteristics of the sample? e.g. demographic data, date	Construction employees aged 17 to 67 years (mean=40, s.d.=11.938), with the majority of participants falling between 17 and 50 years. Most of the participants reported that they were White British (n = 296, 85.1%), spoke English as a first language (n=397, 93.2%) and identified as heterosexual (n = 415, 97.4%)
Data collection		
17. Interview guide	Were questions, prompts, guides provided by the authors? Was it pilot tested?	Questioning guide used for each interview.
18. Repeat interviews	Were repeat interviews carried out? If yes, how many?	No
19. Audio/visual recording	Did the research use audio or visual recording to collect the data?	Interviews were audio-recorded using two Dictaphones
20. Field notes	Were field notes made during and/or after the interview or focus group?	No due to short nature of interviews field notes were not taken but small prompts for the interviewer were taken to encourage engagement of participants
21. Duration	What was the duration of the interviews or focus group?	Approx. 5 to 10 minutes
22. Data saturation	Was data saturation discussed?	Yes
23. Transcripts returned	Were transcripts returned to participants for comment and/or correction?	No – due to the nature of the research. Workplace location and anonymity did not allow for this
Domain 3: analysis and findings		
Data analysis		
24. Number of data coders	How many data coders coded the data?	One – reviewed by a second researcher (CE).
25. Description of the coding tree	Did authors provide a description of the coding tree?	No, however initial coding was informed by the interview guide. Coding was reviewed by a second researcher (CE) for consensus.
26. Derivation of themes	Were themes identified in advance or derived from the data?	Themes were derived from the data
27. Software	What software, if applicable, was used to manage the data?	NVIVO software was used to manage the data
28. Participant checking	Did participants provide feedback on the findings?	No
Reporting		
29. Quotations presented	Were participant quotations presented to illustrate the themes / findings? Was each	Yes

	quotation identified? e.g. participant number	
30. Data and findings consistent	Was there consistency between the data presented and the findings?	Yes
31. Clarity of major themes	Were major themes clearly presented in the findings?	Yes
32. Clarity of minor themes	Is there a description of diverse cases or discussion of minor themes?	Yes
