# Supplementary material for: Accessing Voluntary HIV Testing in the Construction Industry: A Qualitative Analysis of Employee Interviews from the Test@Work Study
Source: Int J Environ Res Public Health. 2021 Apr 15;18(8):4184. doi: 10.3390/ijerph18084184 (PMC8071335; doi:10.3390/ijerph18084184)
Supplement: Supplementary file 1 [file ijerph-18-04184-s001.zip › ijerph-1176120-supplementary/ijerph-1176120-supplementary/Supplementary file S2 - Interview schedule.pdf]

Supplementary file S2: Interview schedule

What are your views about promoting health to people in their place of work?

In what ways, if any, does your workplace support your physical or mental health, or accessing help for health issues?

Can you tell me the reasons why you attended this event today?

What are your views about the health checks event?

Prompts…What was good? What was not so good? What could be done to improve such events in future?

Did you have any tests or check-ups during the event today? Yes/No

Which did you have? Why did you choose to have them (or not)?

How do you feel about the fact that HIV testing was an option in this event?

Did you take an HIV test yourself? Yes/No

If yes, why?
If no, why not?

What type of health promotion activities would you like to see at your workplace in the future?

Thank you for taking part.
